# A care bundle including antenatal corticosteroids reduces preterm infant mortality in Tanzania a low resource country

**DOI:** 10.1371/journal.pone.0193146

**Published:** 2018-03-07

**Authors:** Augustine Massawe, Hussein L. Kidanto, Robert Moshiro, Edna Majaliwa, Flora Chacha, Aisa Shayo, Paschal Mdoe, Prisca Ringia, Mary Azayo, Georgina Msemo, Estomih Mduma, Hege L. Ersdal, Jeffrey M. Perlman

**Affiliations:** 1 Paediatrics and Child Health, School of Medicine, Muhimbili University of Health and Allied Sciences, Dar es Salaam, Tanzania; 2 Obstetrics and Gynecology, Muhimbili University of Health and Allied Sciences, Dar es Salaam, Tanzania; 3 Paediatrics, Catholic University of Health and Allied Sciences-Bugando, Mwanza, Tanzania; 4 Department of Paediatrics, Kilimanjaro Christian Medical Center, Moshi, Tanzania; 5 Obstetrics and Gynecology, Haydom Lutheran Hospital, Haydom, Tanzania; 6 Division of Obstetric Nursing, Weill Bugando Hospital, Mwanza, Tanzania; 7 Reproductive and Child Health Services, Ministry of Health and Social Services, Dar es Salaam, Tanzania; 8 Department of Anaesthesiology and Intensive Care, Stavanger University Hospital, Stavanger, Norway; 9 Department of Pediatrics, Weill Cornell Medicine, New York, NY, United States of America; Center of Pediatrics, GERMANY

## Abstract

**Background:**

Preterm neonatal mortality (NM) has remained high and unchanged for many years in Tanzania, a resource-limited country. Major causes of mortality include birth asphyxia, respiratory insufficiency and infections. Antenatal corticosteroids (ACS) have been shown to significantly reduce mortality in developed countries. There is inconsistent use of ACS in Tanzania.

**Objective:**

To determine whether implementation of a care bundle that includes ACS, maternal antibiotics (MA), neonatal antibiotics (NA) and avoidance of moderate hypothermia (temperature < 36°C) targeting infants of estimated gestational age (EGA) 28 to 34 6/7 weeks would reduce NM (< 7 days) by 35%.

**Methods:**

A Pre (September 2014 to May 2015) and Post (June 2015 to June 2017) Implementation strategy was used and introduced at three University-affiliated and one District Hospital. Dexamethasone, as the ACS, was added to the national formulary in May 2015, facilitating its free use down to the district level.

**Findings:**

NM was reduced 26% from 166 to 122/1000 livebirths (P = 0.005) and fresh stillbirths (FSB) 33% from 162/1000 to 111/1000 (p = 0.0002) Pre versus Post Implementation. Medications including combinations increased significantly at all sites (p<0.0001).

By logistic regression, combinations of ACS, maternal and NA (odds ratio (OR) 0.33), ACS and NA (OR 0.30) versus no treatment were significantly associated with reduced NM. NM significantly decreased per 250g birthweight increase (OR 0.59), and per one week increase in EGA (OR 0.87). Moderate hypothermia declined pre versus post implementation (p<0.0001) and was two-fold more common in infants who died versus survivors.

**Interpretation:**

A low-cost care bundle, ~$6 per patient, was associated with a significant reduction in NM and FSB rates. The former presumably by reducing respiratory morbidity with ACS and minimizing infections with antibiotics. If these findings can be replicated in other resource-limited settings, the potential for further reduction of <5 year mortality rates becomes enormous.

## Introduction

One-week neonatal mortality still contributes significantly to the under-5-year mortality rates in Tanzania. [1, 2] Major contributing causes include birth asphyxia (BA), prematurity and infection. The number of preterm infant deaths in Tanzania remains substantial at approximately 9500 annually. [2, 3] We demonstrated several years ago in a pilot study that implementation of the Helping Babies Breathe (HBB) program was associated with a significant 47% reduction in early neonatal mortality (NM), i.e. less than 24 hours; this reduction in mortality extended to infants less than 34 weeks gestation. [4]

Tanzania has limited capacity to continuously monitor either heart rate or respirations and/or manage preterm infants with any respiratory distress in the newborn/preterm nursery. Specifically, the capacity to deliver continuous positive airway pressure (CPAP), to intubate and administer surfactant as indicated, and support breathing with positive pressure ventilation is inadequate. Moreover, antibiotics are only initiated when the preterm infant becomes symptomatic.

Given these limitations in care, we focused our attention towards a seemingly low-cost, evidence-based preventative approach, which is part of standard practice in resource-replete countries. These strategies include the administration of ACS to mothers in preterm labour, [5, 6] maternal antibiotics when in active labour, [7, 8] immediate stabilization/resuscitation of the newborn [9] including avoidance of moderate hypothermia (<36°C), [10] and early neonatal antibiotics which we consider to be crucial first steps to reducing the burden of preterm infant mortality. These efforts had to be partially reset following publication of a cluster randomized study in low and middle-income countries comparing ACS versus standard care administered to preterm infants of estimated gestational age (EGA) 24 to 37 weeks for the reduction of NM. The study showed, among the whole population, that both 28-day NM and suspected maternal infection were significantly more common in the intervention group. [11] There were several factors that limited relevance of the findings to Tanzania and other resource-limited countries. First, those in the intervention group were less likely to be delivered in a hospital. Second, there was a failure to address issues such as the immediate application of HBB upon delivery, as well as the avoidance of moderate hypothermia, which has previously been shown to have a dose-dependent relationship with mortality. [12, 13] These were critical factors in our estimation, contributing to the “unanticipated” findings in this report. [14] Moreover, as noted previously, all of the above interventions constitute standard practice in resource-replete countries, and components of the package, including ACS administration, were already in use in some of the referral hospitals.

After much national debate, the Ministry agreed to evaluate the potential benefit of the care package which in addition included application of HBB principles and the avoidance of moderate hypothermia in a pilot rollout as a precursor to potential national dissemination. The objective of this pilot initiative was to determine whether implementation of the care package including avoidance of moderate hypothermia to preterm infants of estimated EGA 28 to 34 6/7 weeks, would be associated with a 35% reduction in NM ≤ 7 days.

## Materials and methods

### Implementation of the care package

This pilot initiative of implementing the care bundle has been supported by the Ministry of Health and Social Welfare. Four hospitals, designated as “study sites.” included Muhimbili National Hospital (MNH), Kilimanjaro Christian Medical Centre (KCMC), Bugando Medical Centre (BMC) and Haydom Lutheran Hospital, were selected to evaluate the care bundle. The process to implementation proceeded as outlined next.

### National conference–prelude to development of a care bundle

A national conference was convened in Dar-es-Salaam March in 2014 and was attended by the Ministry of Health and Social Welfare, Pediatricians, Obstetricians, and Midwives representing all regions in Tanzania, as well as Non-Government organizations including USAID, Save the Children, UNICEF, the World Health Organization (WHO) and the Laerdal Foundation for Acute Medicine. The care bundle that was discussed included ACS administration to mothers of fetuses of GA 28 to 34 6/7 weeks, maternal antibiotics when in labour, implementation of HBB at delivery, avoidance of moderate hypothermia starting at delivery, and early administration of neonatal antibiotics. Thus infants delivered following a preterm labour were empirically treated for 48 hours if asymptomatic, and longer if there clinical signs, i.e. tachypnea, retractions, etc. Exceptions included delivery secondary to maternal conditions (e.g. pre-eclampsia). The antibiotic strategy was based on the fact that most cases of chorioamnionitis are asymptomatic, i.e. noted on histology only. Moreover, blood cultures and inflammatory markers are rarely obtained to evaluate for infection, and only then at the time of symptoms.

The estimated cost of the care bundle medications was $6 to $7 per mother/infant pair.

In June 2015, national guidelines for the use of ACS was prepared, followed by its addition to the national formulary as an essential medication. This action facilitated the free use of ACS down to the district hospital level. This strategy of making ACS freely available was consistent with the suggestion by a Working Group for the UN Commission of Life Saving Commodities. [15]

### Development of educational tools

**Fig 1:**
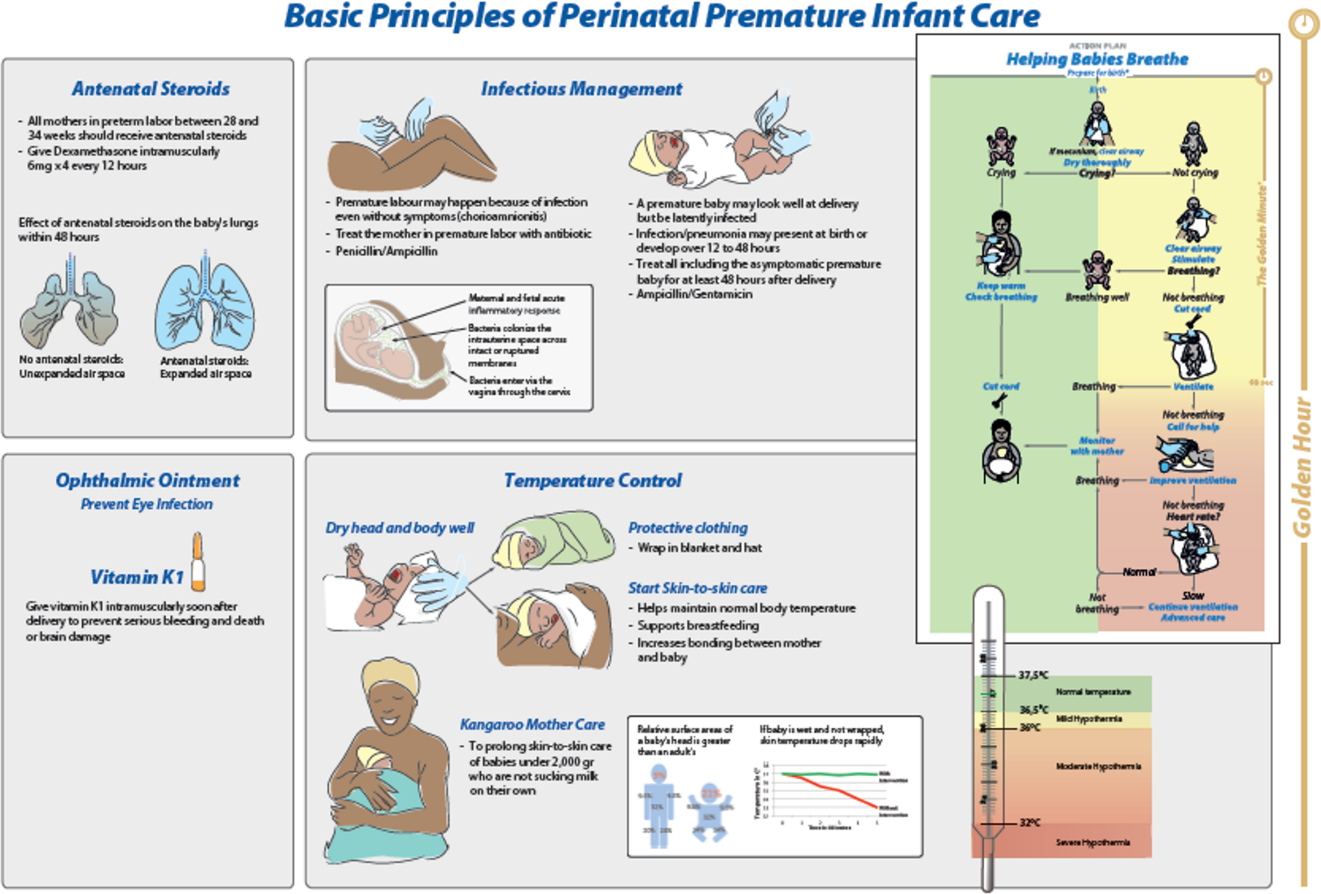
Flow diagram depicting the interventions to be applied in the bundle of care treatment strategy.

A 14-page educational handbook was developed that outlined the proposed interventions, including the rational for each strategy, as well as dosing regimen for each medication. This included Dexamethasone (6 mg intramuscularly x 4 doses every 12 hours) which was the specified ACS used, Penicillin and/or Ampicillin were the maternal antibiotics, and Ampicillin (50 mg/kg every 12 hours) and Gentamicin (4 mg/kg every 24 hours) were administered intravenously to the baby. The WHO ten-step strategy for maintaining temperature was emphasized. [15] A flow diagram poster depicting the interventions was also developed which supplemented the educational handout (Fig 1). The poster was placed in the delivery room resuscitation area at each hospital.

Importantly, the care bundle, including avoidance of moderate hypothermia, was conceived as a bridge between labour and delivery, the first postnatal hour, and subsequent management based on the WHO Essential Newborn Care which is practiced in almost all hospitals. [16, 17]

There was limited capacity to apply CPAP and no preterm infant was intubated.

### Implementation team

HK was designated as the primary investigator of the project. A dedicated Pediatrician, Obstetrician and Midwife was appointed at each site to facilitate implementation of the care bundle. In general, almost all vaginal deliveries are managed by a midwife. All midwives at the study hospitals are trained in the HBB program. A data recorder was assigned at each hospital. Review sessions were conducted by JMP at the hospitals in August 2014, March 2015, August 2015, March 2016, August 2016 and March 2017. In addition, all team members participated at a bi-annual data monitoring meeting in Dar-es Salaam every March and August, where ongoing data related to the project was reviewed, implementation barriers identified and rectified where possible.

### Program evaluation

#### Data monitoring

**Fig 2:**
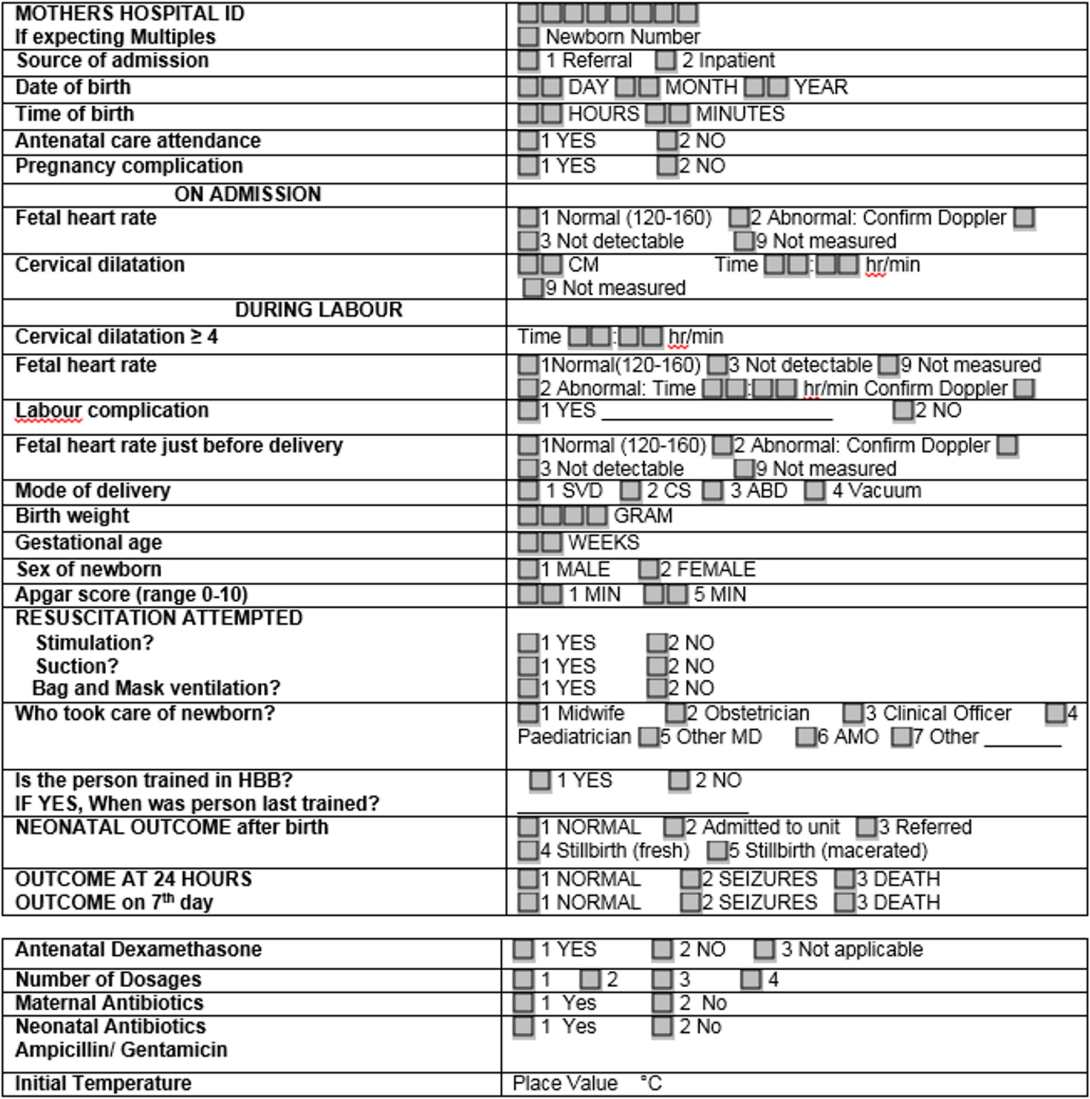
Data collection sheet.

A dedicated computer close to every labour ward was used for data entry and subsequent transmission of data to a central repository in the Health Ministry in Dar es Salaam. A data collection form, which included core and desired elements developed for the HBB rollout in Tanzania, was supplemented by data points specific to the project. This included ACS use, number of doses, maternal and neonatal antibiotic use, and initial neonatal temperature. (Fig 2)

Each hospital started collecting Pre Implementation baseline data in September 2014 with the implementation process initiated in June 2015. Data were analyzed by a data analyst (HK) and technical consultant (JMP), and evaluated every six months by the study participants. Consistency checks of the data were performed intermittently by HK and every six months by the technical consultant at the primary sites. There were no formal stopping rules.

#### Definitions

Gestational age (GA) was based on self-report of the last normal menstrual period and/or fundal height, the latter the distance from symphysis pubis to the uterine fundus in the middle of the woman’s abdomen, as is the standard practice in Tanzania. [18] Preterm infants of presumed EGA 28 to 34 6/7 weeks were eligible to receive the care package. Birth weight (BW) cutoff for live births was ≥ 750 grams. Fresh stillbirth (FSB) was defined as an Apgar score = 0 at both 1 and 5 minutes with intact skin and suspected death during labour/delivery, and of birth weight >1000 grams.

#### Study Goals

The primary goal was to reduce the rate of Preterm mortality in the first 7 days after birth by 35%.

#### Statistical analysis

A power analysis was calculated to detect a 35% reduction in NM. This was based on preliminary pre implementation data indicating a NM rate ≤ 7 days of 166/1000 live births for infants < 35 weeks EGA. This indicated that approximately 545 babies pre versus post implementation were needed with 80% power, and with use of a two-tailed test at a significance level of 5%.

Additional subgroup analysis was undertaken to determine the potential impact of the care bundle on infants of EGA 28 to 30 6/7 weeks and those of 31 to 34 6/7 weeks.

Analysis has been performed using Statistical Package for Social Sciences (SPSS) 22; and included descriptive statistics, chi square analysis, t tests and relative risk (RR) calculations. Odds ratios (OR) were calculated from the logistic regression analysis. A bivariate multiple logistic regression was developed to estimate effects of administration of ACS, maternal and neonatal antibiotics on predicting 7-day mortality while controlling for BW, EGA, source of admission, delivery mode and gender. Due to the high percentage of administration of combinations of ACS, maternal and neonatal antibiotics (70%) in the observed population, a new variable with 8 scenarios of either combination of medicines or singular use versus no medications which was dummy coded and served as the reference scenario. The data was analyzed for the entire cohort followed by subgroup analysis for preterm infants of EGA 28 to 30 6/7 weeks and 31 to 34 6/7 weeks.

All data are presented as mean ±standard deviation unless as otherwise stated.

#### Ethical considerations

Implementation of the care bundle received ethical clearance from the National Institute of Medical Research of Tanzania. NIMR/HQ/R.8c/Vol.11/677. Since components of the care bundle were already being clinically utilized to some extent at all hospitals, and are considered standard practice in the resource replete settings, informed consent was not sought.

## Results

**Fig 3:**
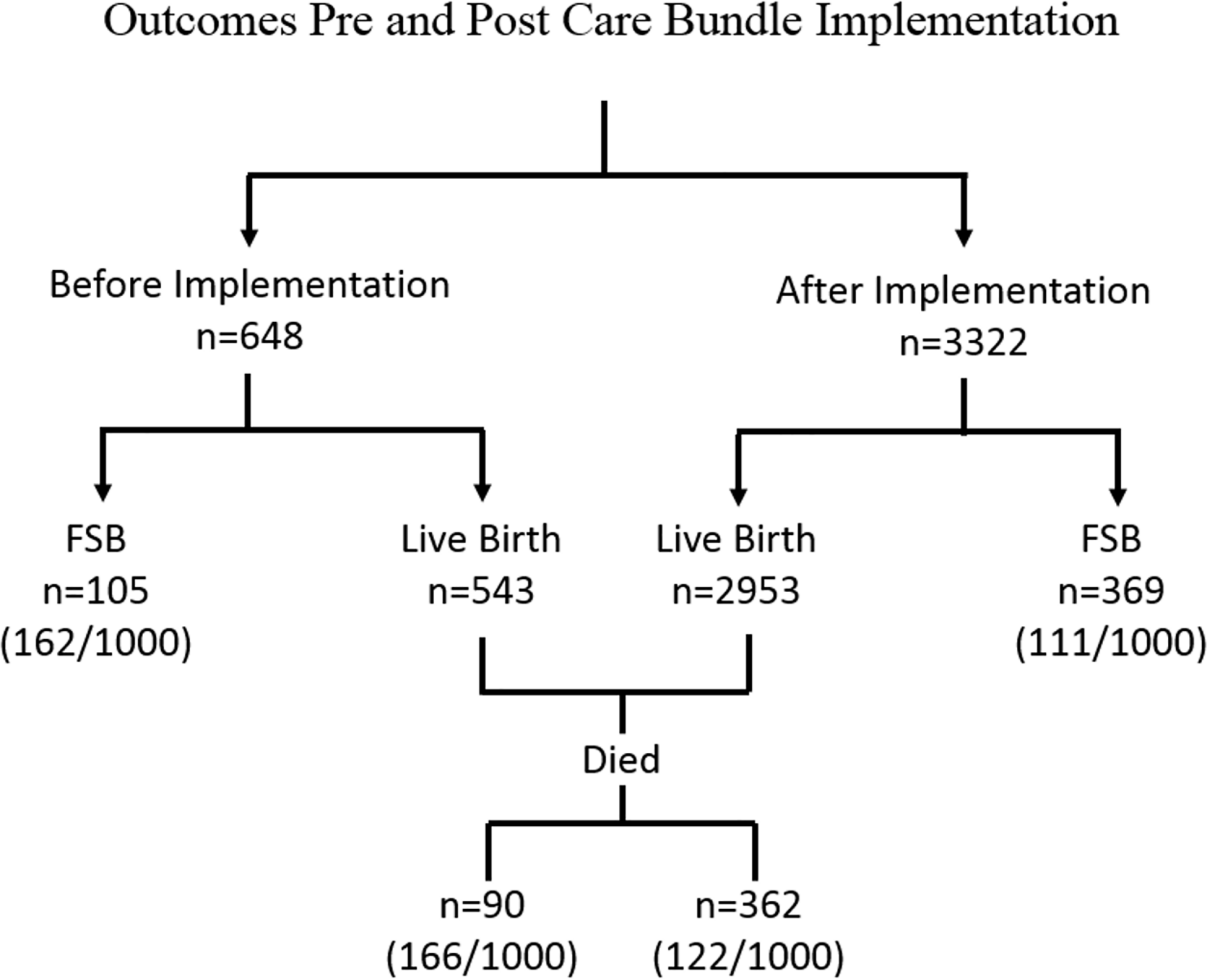
Outcomes pre and post implementation.

Between September 2014 and June 2017 there were a total of 3496 preterm infants born; 543 were pre-implementation (September 2014 to May 2015) of whom 90 (166/1000) died. During implementation (June 2015 to June 2017) there were 2953 preterm infants born of whom 362 (122/1000) died. (Fig 3) This reflects a 26% reduction, i.e. RR 0.74 (95% confidence interval (CI) 0.60–0.91) (p = 0.005) pre vs post implementation. (Fig 3). There were 105 FSB (162/1000) pre and 369 (111/1000) post implementation which represents a 33% reduction, i.e. RR 0.67 (CI 0.54–0.82) (p = 0.0002).

The impact of implementation of the care bundle on death and fresh stillbirth rates for the four hospitals is shown in Table 1. Note the significant reduction in NM at MNH, RR 0.58 (0.42–0.81) (p = 0.0001) only, as well as a reduction in FSB rates RR 0.46 (0.36–0.59) (p<0.0001). At BMC there was a significant reduction in FSB rates 0.26 (0.12–0.0.58) (p = 0.0009). There were no differences in NM or FSB rates at the other hospitals.

**Table 1:** Neonatal mortality and fresh stillbirths at the four hospitals, pre and post bundle of care implementation.

Hospital	Neonatal Mortality			Fresh Still Births		
	Pre-Implementation	Post- Implementation	RR, 95% CI, P-Value	Pre-Implementation	Post- Implementation	RR, 95% CI, P-Value
MNH	33/159 (20.7%)	271/2222 (12.2%)	0.58 (0.42–0.81) P = 0.001	58/217 (26.7%)	311/2533 (12.3%)	0.46 (0.36–0.59) P<0.0001
KCMC	27/205 (13.1%)	54/388 (13.9%)	1.05 (0.68–1.62) P = 0.80	20/205 (9.7%)	43/431 (9.9%)	1.12 (0.67–1.86) P = 0.65
BMC	18/118 (15.2%)	27/261 (10.3%)	0.67 (0.38–1.18) P = 0.17	17/135 (12.6%)	9/270 (3.3%)	0.26 (0.12–0.58) P = 0.0009
Haydom	12/61 (19.7%)	10/82 (12.2%)	0.61 (0.28–1.34) P = 0.22	10/71 (14.1%)	6/88 (6.8%)	0.48 (0.12–1.26) P = 0.13
Totals	90/543 (16.6%)	362/2953 (12.2%)	0.74 (0.60–0.91) P = 0.005	105/648 (16.2%)	369/3322 (11.1%)	0.67 (0.54–0.82) P = 0.0002

MNH = Muhimbili National Hospital, KCMC = Kilimanjaro Christian Medical Centre, BMC = Bugando Medical Center. RR = Relative Risk, CI = Confidence Interval

### General characteristics and medication use pre versus post implementation

Infants were of comparable BW (p = 0.10) but lesser GA (p<0.0001) pre versus post implementation (Table 2). There were no differences related to gender and mode of delivery. However, there were more referrals post implementation (p = 0.0001) (Table 2). The use of maternal antibiotics, ACS, neonatal antibiotics and a combination of medication increased with implementation (p<0.0001 for all). (Table 2). Temperature measurements increased more than three-fold pre versus post implementation (p<0.0001). The number of infants with moderate hypothermia decreased two-fold pre versus post implementation (p<0.0001). Conversely the number of infant with a temperature in the normal range increased more than three-fold pre versus post implementation (p<0.0001). (Table 2)

**Table 2:** General characteristics of infants before and after implementation.

Category	Pre-Implementation n = 543	Post- Implementation n = 2953	P-Value
Birth Weight (Grams)*	1684 ± 420	1716 ± 424	0.10
Gestational Age (Weeks)	31.17 ± 2.0	31.66 ± 1.99	< 0.0001
Inborn/Referral	320/212	1156/1538	< 0.0001
Gender (Male/Female)	261/282	1453/1495	0.60
Mode of Delivery (Vaginal/C-Section/Vacuum)	279/244/17	1350/1379/218	0.10
Maternal Antibiotics	167 (30.8%)	2071 (70.1%)	< 0.0001
Antenatal Corticosteroids	185 (34%)	2064 (69.9%)	< 0.0001
Neonatal Antibiotics	152 (28%)	2395 (81.1%)	< 0.0001
ACS + Maternal + Neonatal Antibiotics	152 (28%)	1712 (58%)	< 0.0001
Temperature Measurements	152 (28%)	2395 (82%)	< 0.0001
Moderate (33.0–35.9°C) Mild (36.0–36.4°C) Normal (36.5–37.5°C)	42 (27.6%) 86 (56.6%) 24 (15.8%)	317 (13.3%) 825 (34.6%) 1243 (52.1%)	< 0.0001

ACS = Antenatal Corticoid Steroids

*Birthweight missing value n = 7. Gender missing values n = 5, Inborn/Referral missing values (n = 270) Mode of delivery missing values n = 9

Infants 750-1000g Pre versus Implementation n = 25 (4.6%) vs n = 95 (3.2%), p = 0.10.

Infants <28 weeks gestational age Pre vs Implementation n = 7 (1.2%) versus n = 8 (0.27%) p = 0.0007. Data analyzed with Students t test and Person Chi Square analysis.

### The general characteristics and medication use at the four hospitals pre versus post implementation (Table 3)

There were differences in BW and GA at MNH (lower) (p< 0.0001), BW at KCMC (greater) (p = 0.01) and BW at Haydom (lower) (p< 0.0001) pre versus post implementation. There were differences in GA at KCMC, BMC and Haydom. There were less referrals post implementation at BMC (p = 0.0002) and more males versus females at Haydom (p = 0.0007) post implementation. There were no differences in mode of delivery at any site. All medication increased significantly at all sites, except at KCMC where there were no differences in ACS use pre versus during implementation, i.e. 53.7 vs 57% (p = 0.40).

**Table 3:** General characteristics and medication use at the four hospitals pre/post implementation.

	Muhimbili National Hosp.			KCMC			Bugando			Haydom		
	Pre-I	Post-I	P Value	Pre-I	Post-I	P Value	Pre-I	Post-I	P Value	Pre-I	Post-I	P Value
	159	2222		205	388		118	261		61	75	
Birthweight (Grams)	1541 ± 322	1711 ± 416	<0.0001	1853 ± 484	1756 ± 457	0.016	1621 ± 339	1601 ± 334	0.59	1736 ± 373	1989 ± 31.6	<0.0001
Gestational Age (Weeks)	31.1 ± 2.1	31.9 ± 1.9	<0.0001	31.6 ± 2.1	31.69 ± 2	0.60	30.3 ± 1.36	30.28 ± 1.60	0.90	31.8 ± 1.9	31.6 ± 2.0	0.55
Inborn/ Referred	53/ 106	784/ 1296	0.30	102/ 103	180/ 208	0.43	114/ 4	158/ 29	0.0002	50/ 11	60/ 15	0.77
**Mode of** **Delivery**												
Vaginal	68	995	0.49	101	165	0.21	64	137	0.91	46	53	0.35
C-Section	80	1037		97	200		52	117		15	25	
Vacuum	8	184		7	23		2	7		0	4	
**Gender**												
Male/ Female	73/ 86	1048/ 1170	0.74	102/ 103	222/ 166	0.08	60/ 58	130/ 131	0.85	26/ 35	53/ 22	0.002
**Medications**												
Maternal Antibiotics	42 (26.4%)	1674 (75.3%)	<0.0001	104 (50.7%)	295 (76%)	<0.0001	14 (11.9%)	78 (29.9%)	<0.0001	7 (11.5%)	24 (29%)	0.01
ACS	28 (17.6%)	1629 (73.3%)	<0.0001	110 (53.7%)	222 (57%)	0.40	45 (38.1%)	195 (74.7%)	<0.0001	2 (3.3%)	18 (22%)	<0.0001
Neonatal Antibiotics	2 (1.3%)	1892 (85.1%)	<0.0001	107 (52%)	298 (76.8%)	<0.0001	32 (27.1%)	181 (69%)	<0.0001	18 (29.5%)	51 (62%)	0.0001
Temperature Measured	0	1780 (80%)		81 (39.5%)	297 (76.5%)	<0.0001	58 (49%)	258 (99%)	<0.0001	13 (21.3%)	50 (61%)	<0.0001

I = Implementation, C-Section = Cesarean section, ACS = Antenatal Corticosteroids. KCMC = Kilimanjaro Christian Medical Center.Data were analyzed with Pearson Chi Square and student t tests where appropriate.

### Characteristics of infants who died versus infants who survived (Table 4)

Infants who died versus those who survived were of a lesser BW and GA (p<0.0001 for both), were more likely to be referred (p < 0.0001), and delivered by a vacuum extraction (p = 0.007). There were no differences related to sex. The administration of maternal antibiotics (p = 0.01), ACS, neonatal antibiotics and a combination of ACS, maternal and neonatal antibiotics were all less in those who died versus survivors (p<0.0001 for all). Infants who died versus survivors were two-fold more likely to present with moderate hypothermia (p<0.0001) and less likely to have a temperature in the normal range (p = 0.003). (Table 4)

**Table 4:** General characteristics of infants <35 weeks who survived versus died.

	Survived n = 3044	Death n = 452	p
Birthweight (grams)*	1761 ± 398	1379 ± 409	< 0.0001
Gestational Age (weeks)	31.98 ± 1.9	30.6 ± 2.1	< 0.0001
Source of Admission–Inborn	1330 (47%)	146 (35%)	< 0.0001
Gender: Male	1493 (49.1%)	221 (49%)	0.965
Mode of Delivery			
Vaginal	1409(46.4%)	220 (48.7%)	0.66
Cesarean Section	1430 (47.1%)	193 (42.7%)	0.27
Vacuum	196 (6.5%)	39 (8.6%)	0.007
Maternal Antibiotics given	1987 (65.3%)	251 (55.5%)	0.01
Antenatal Corticosteroids given	2033 (66.8%)	216(47.8%)	< 0.0001
Neonatal Antibiotics given	2236 (76.7%)	245 (52.8%)	< 0.00001
Antenatal Corticosteroids and Maternal and Neonatal Antibiotics	1577 (51.8%)	133 (29.4%)	< 0.0001
Temperature measurement**	2248 (73.7%)	299 (66.2%)	0.0001
Hypothermia:			
Moderate 33.0–35.9°C Mild 36.0–36.4°C Normal 36.5–37.5°C	282 (12.6%) 813 (36.3%) 1144 (51.1%)	77 (25.8%) 98 (32.9%) 123 (41.3%)	< 0.0001 0.25 0.003

Birthweight * Missing Values n = 7

Temperature measurements** missing Pre-implementation n = 796, Post-implementation n = 153 (p<0.0001).

Data were analyzed with Pearson Chi Square and student t tests where appropriate.

### Impact of ACS dosing on NM rates

There was a dose dependent effect of ACS on NM for the entire cohort (Table 5). Comparing no ACS exposure versus a full course (3/4 doses), there was a significant reduction in NM from 18.9% (no ACS) to 7.6% (3/4 doses) (RR 0.48 (CI 0.37–0.61), p<0.0001. Comparing no ACS exposure versus a partial course (1/2 doses), there was a significant reduction in NM from 18.9% (no ACS) to 12.6% (partial course) (RR 0.66 (CI .0.54–0.81) (p = 0.0001). Comparing partial versus a complete course there is a significant reduction in NM from 12.6% (partial) to 7.6% (complete course) (RR 0.60 (CI 0.47–0.78 (p = 0.0001).

**Table 5:** Outcome of preterm infants expected to 1/2 (partial) or 3/4 (complete) doses of dexamethasone versus no treatment.

	No Doses	1/2 Doses	3/4 Doses
**Survived**	817*#	1030	1059
**Died**	191 (18.9%)	149 (12.6%)**	88 (7.6%)
**Total**	1008	1179	1147

Missing doses n = 162

* Difference in outcome for infants who died (none vs 3/4 doses) p<0.0001

# Difference in outcome for infants who died (none vs 1/2 doses) p = 0.0001

** Difference in outcome for infants (1/2 doses vs 3/4 doses) p = 0.0001

### Stepwise logistic regression analysis

Stepwise logistic modelling included BW in increments of 250 grams, GA in increments of one week, inborn versus referral, mode of delivery, sex and medications either singularly or in combination. Initial temperature was not included as a predictor due to 27.1% of missing values.

Significant predictors of a reduction in NM were combination therapy, i.e. ACS and neonatal antibiotics (OR 0.303, p = 0.0001) and ACS, maternal and neonatal antibiotics (OR 0.332, p = 0.0001), inborn cases (OR 0.57, p = 0.0001), increased BW (OR 0.593) and GA (OR 0.877, both p = 0.0001). Predictors associated with increased NM risk, included ACS and maternal antibiotics (OR 2.825, p = 0.0001) and ACS only (OR 1.796, p = 0.071). (Table 6)

**Table 6:** Stepwise logistic regression Analysis for estimating effects of administration of antenatal corticosteroids (ACS), maternal & neonatal antibiotics in combination or singularly for predicting death in preterm infants while controlling for BW, GA, source of admission, mode of delivery and gender.

		B	SE	Sig	Exp(B)	95% CI for Exp(B)	
						Lower	Upper
Step 3	BW per 250 g	-0.523	0.042	0.0001	0.593	0.546	0.643
	Gestational Age per week	-0.131	0.031	0.0001	0.877	0.825	0.933
	Inborn	0.562	0.125	0.0001	0.570	0.446	0.729
	ACS+ Maternal+ Neonatal Antibiotics	-1.103	0.154	0.0001	0.332	0.245	0.449
	ACS + Neonatal Antibiotics	-1.194	0.234	0.001	0.303	0.192	0.479
	ACS +Maternal Antibiotics	1.050	0.326	0.001	2.825	1.507	5.414
	ACS only	0.586	0.324	0.071	1.796	0.952	3.399
	Maternal and Neonatal Antibiotics	-0.309	0.232	0.182	0.734	0.466	1.156
	Maternal Antibiotics	-0.016	0.225	0.932	0.984	0.633	1.530
	Neonatal Antibiotics	-0.246	0.262	0.346	0.782	0.468	1.305
	Constant	4.945	0.920	0.0001	140.491		

BW = Birth weight, GA = Gestational Age, ACS = Antenatal Corticosteroids

Infants with missing values (n = 288) for predictors were excluded. Initial temperature was not included as a predictor due to 27.1% of missing values.

### Impact of the care bundle on preterm infants of EGA 28 to 30 6/7 weeks and 31 to 34 6/7 weeks (Table 7)

NM for infants 28 to 30 6/7 weeks was 45/230 (19.6%) versus 198/884 (22.4%), (RR 1.14 (CI 0.85–1.52) (p = 0.35), and for infants 31 to 34 6/7 weeks 45/313 (14.4%) versus 164/2069 (7.9%) RR 0.55 (CI 0.40–0.75) (p = 0.0001) pre versus post implementation respectively. The subgroup analysis for infants who survived versus those who died was similar to the overall cohort of infants. (Table 7). Specifically infants who died versus those who survived were of a lesser BW and GA for both subgroups (p<0.0001 for both), were more likely to be referred for both subgroups i.e. infants 28–30 6/7 weeks (p < 0.0001) and 31 to 34 6/7 weeks (p = 0.01). There were no differences related to sex. The administration of maternal antibiotics was not different for infants 28 to 30 6/7 weeks (p = 0.45), but infants 31 to 34 6/7 weeks who survived were more likely to receive maternal antibiotics (p = 0.003). Administration of ACS only, neonatal antibiotics only, and a combination of ACS, maternal and neonatal antibiotics were all less in those who died versus survivors (p<0.0001) for both subgroups. Moderate hypothermia was 1.5 fold more likely in infants of 28 to 30 6/7 weeks (p = 0.002), and 2.5 fold more likely in infants 31 to 34 6/7 weeks (p = 0.0001) who died versus those who survived.

**Table 7:** General characteristics of Infants 28–30 6/7 weeks vs. 31–34 6/7 weeks who survived versus died.

	28–30 6/7 Weeks n = 1114			31–34 6/7 Weeks n = 2382		
	Survived n = 871	Died n = 243	p	Survived n = 2173	Died n = 209	p
Birthweight (grams)^1^	1558 ± 406	1242 ± 366	< 0.00001	1842 ± 372	1540 ± 390	< 0.00001
Gestational Age (weeks)	29.25 ± 0.88	28.87 ± 1.09	< 0.00001	32.86 ± 1.04	32.48 ± 1.047	< 0.0001
Inborn^2^	463 (57.4%)	81 (37.3%)	< 0.00001	867 (43.2%)	65 (33.7%)	0.01
Gender: Male^3^	433 (49.7%)	122 (50.2%)	0.89	1060 (51.1%)	109 (52.4%)	0.72
Maternal Antibiotics given	475 (54.5%)	126 (51.9%)	0.45	1512 (69.6%)	125 (59.8%)	0.003
ACS	547 (62.8%)	119 (49%)	0.0001	1486 (68.4%)	97 (46.4%)	< 0.0000
Neonatal Antibiotics given	603 (69.2%)	129 (53.1%)	< 0.00001	1733 (79.8%)	116 (55.5%)	< 0.0000
ANS and Maternal and Neonatal Antibiotics	371 (42.6%)	75 (30.9%)	0.003	1206 (55.5%)	58 (27.8%)	= 0.0001
Temperature measurement^4^	612 (70.2%)	159 (65.4%)	0.15	1627 (74.8%)	139 (66.5%)	0.003
Hypothermia:						
Moderate 33.0–35.9°C Mild 36.0–36.4°C Normal 36.5–37.5°C	88 (14.4%) 241 (39.4%) 283 (46.2%)	39 (24.5%) 51 (32.1%) 69 (43.4%)	0.002 0.09 0.52	194 (11.9%) 572 (35.2%) 861 (52.9%)	38 (27.3%) 47 (33.8%) 54 (38.8%)	0.0001 0.74 0.001

ACS = Antenatal corticosteroids. Data were analyzed with Pearson Chi Square and Student t tests where appropriate.

Birthweight^1^ missing values n = 7

Inborn^2^ missing values n = 270

Gender^3^ missing values n = 5

Temperature^4^ measurements missing values n = 949 (27.1%) Temperature categories excluded n = 10 that had temperature >37.5°C

### Stepwise logistic regression analysis for infants 28 to 30 weeks and 31 to 34 6/7 weeks EGA (Tables 8 and 9)

Stepwise logistic modelling for both subgroups was similar to the overall cohort. Significant predictors of a reduction in NM were combination therapy, i.e. ACS and neonatal antibiotics, i.e. for infants 28 to 30 6/7 weeks (OR 0.253, p = 0.001) and infants 31 to 34 6/7 weeks (OR 0.384, p = 0.003) as well as for combined ACS, maternal and neonatal antibiotics, i.e. for infants 28 to 30 6/7 weeks (OR 0.397, p = 0.0001) and for infants 31 to 34 6/7 weeks (OR 0.282, p = 0.0001). Additional predictors of reduced NM included inborn cases for 28 to 30 6/7 weeks only (OR 0.45, p = 0.0001), increased BW (OR 0.59) (p = 0.0001) for both groups and GA (OR 0.765) (p = 0.001) for infants 31 to 34 6/7 weeks only. Predictors associated with increased NM risk, included ACS and maternal antibiotics (OR 3.25, p = 0.01) and ACS only (OR 1.79, p = 0.071) in infants of GA 31 to 34 6/7 weeks. (Table 9)

**Table 8:** Stepwise logistic regression analysis for estimating effects of administration of antenatal corticosteroids (ACS), maternal & neonatal antibiotics in combination or singularly for predicting death in p[reterm infants while controlling for BW, GA, source of admission, mode of delivery and gender for infants 28 to 30 6/7 weeks GA.

	B	SE	Sig	Exp(B)	95% CI for Exp(B)	
					Lower	Upper
BW per 250 g	-0.515	0.063	0.0001	0.597	0.528	0.675
Gestational Age per week	-0.115	0.089	0.197	0.892	0.749	1.061
Inborn	-0.797	0.177	0.0001	0.451	0.319	0.638
ACS+ Maternal+ Neonatal Antibiotics	-0.923	0.215	0.0001	0.397	0.261	0.606
ACS + Neonatal Antibiotics	-1.372	0.336	0.001	0.253	0.131	0.490
ACS +Maternal Antibiotics	0.610	0.483	0.206	1.84	0.715	4.72
ACS only	0.181	0.447	0.686	1.198	0.499	2.876
Constant	4.997	2.496	0.045	148.004		

BW = Birth weight, GA = Gestational Age ACS = Antenatal Corticosteroids

Infants with missing values (n = 288) for predictors were excluded. Initial temperature was not included as a predictor due to 27.1% of missing values.

**Table 9:** Stepwise logistic regression analysis for estimating effects of administration of antenatal corticosteroids (ACS), maternal & neonatal antibiotics in combination or singularly for predicting death in preterm infants while controlling for BW, GA, source of admission, mode of delivery and gender for infants 31 to 346/7 weeks.

	B	SE	Sig	Exp(B)	95% CI for Exp(B)	
					Lower	Upper
BW per 250 g	-0.527	0.057	0.0001	0.590	0.528	0.660
Gestational Age per week	-0.268	0.078	0.001	0.765	0.657	0.891
Inborn	-0.295	0.177	0.095	0.745	0.527	1.053
ACS+ Maternal+ Neonatal Antibiotics	-1.267	0.227	0.0001	0.282	0.181	0.439
ACS + Neonatal Antibiotics	-0.957	0.327	0.003	0.384	0.202	0.728
ACS +Maternal Antibiotics	1.468	0.466	0.011	3.25	1.305	8.102
ACS only	1.179	0.324	0.071	1.796	0.952	3.399
Constant	9.76	2.49	0.0001	17332.5		

BW = Birth weight, GA = Gestational Age ACS = Antenatal Corticosteroids

### Compliance with the implementation plan

**Fig 4:**
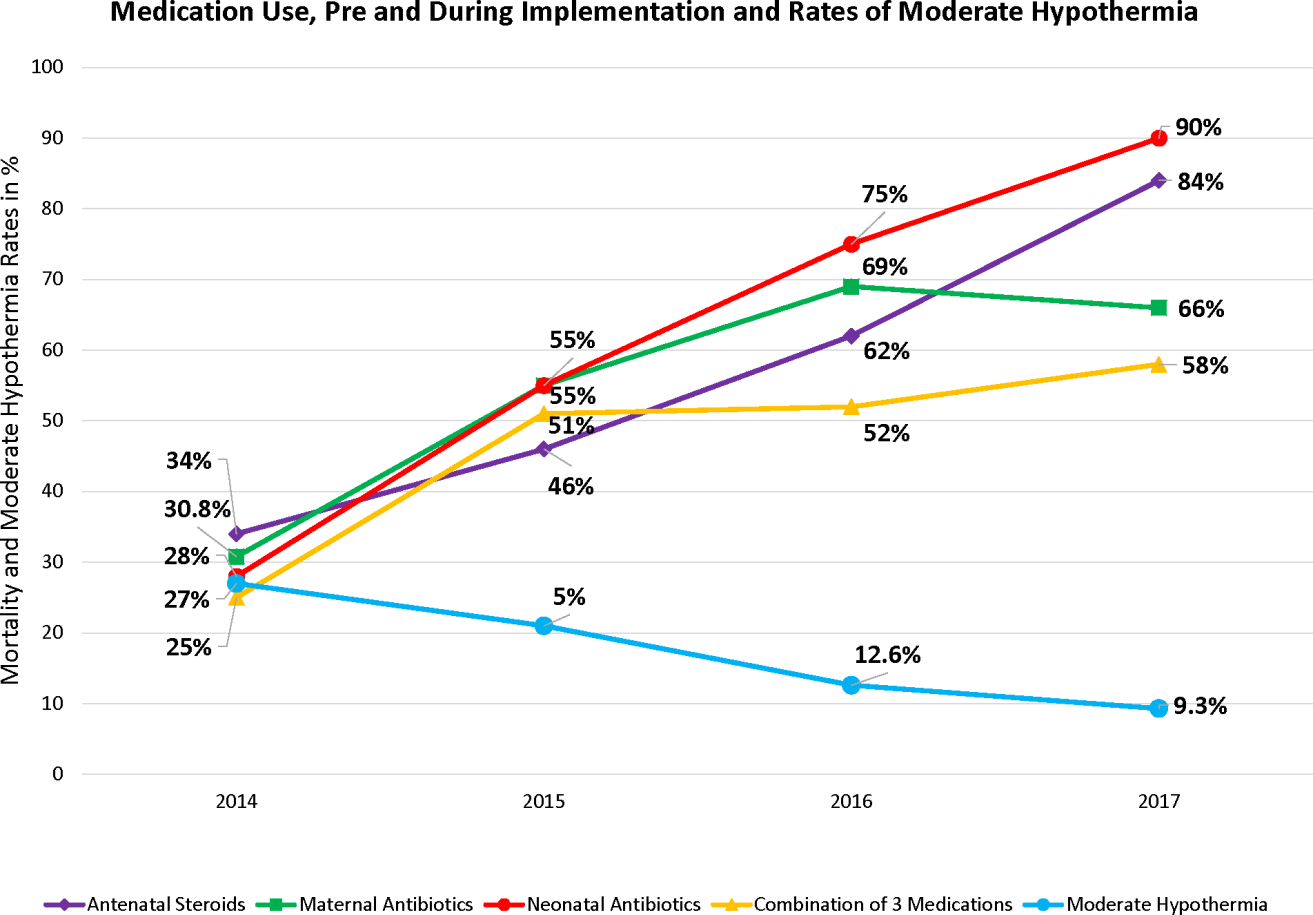
Use of medication either singularly or in combination from September 2014 through June 2017. NM progressively decreased over the comparable time period concurrent with the increased use of medication. (Fig 5).

There was a significant increase in use of all medications over time. The only exception was for ACS use at KCMC, where there was no difference pre versus post implementation. By June 2017, Neonatal Antibiotics had increased to 90%, ACS to 84%, Maternal Antibiotics to 66% and a combination of medications to 58%. Over the corresponding time frame, moderate hypothermia declined three-fold from 27% to 9.3%. (Fig 4)

**Fig 5:**
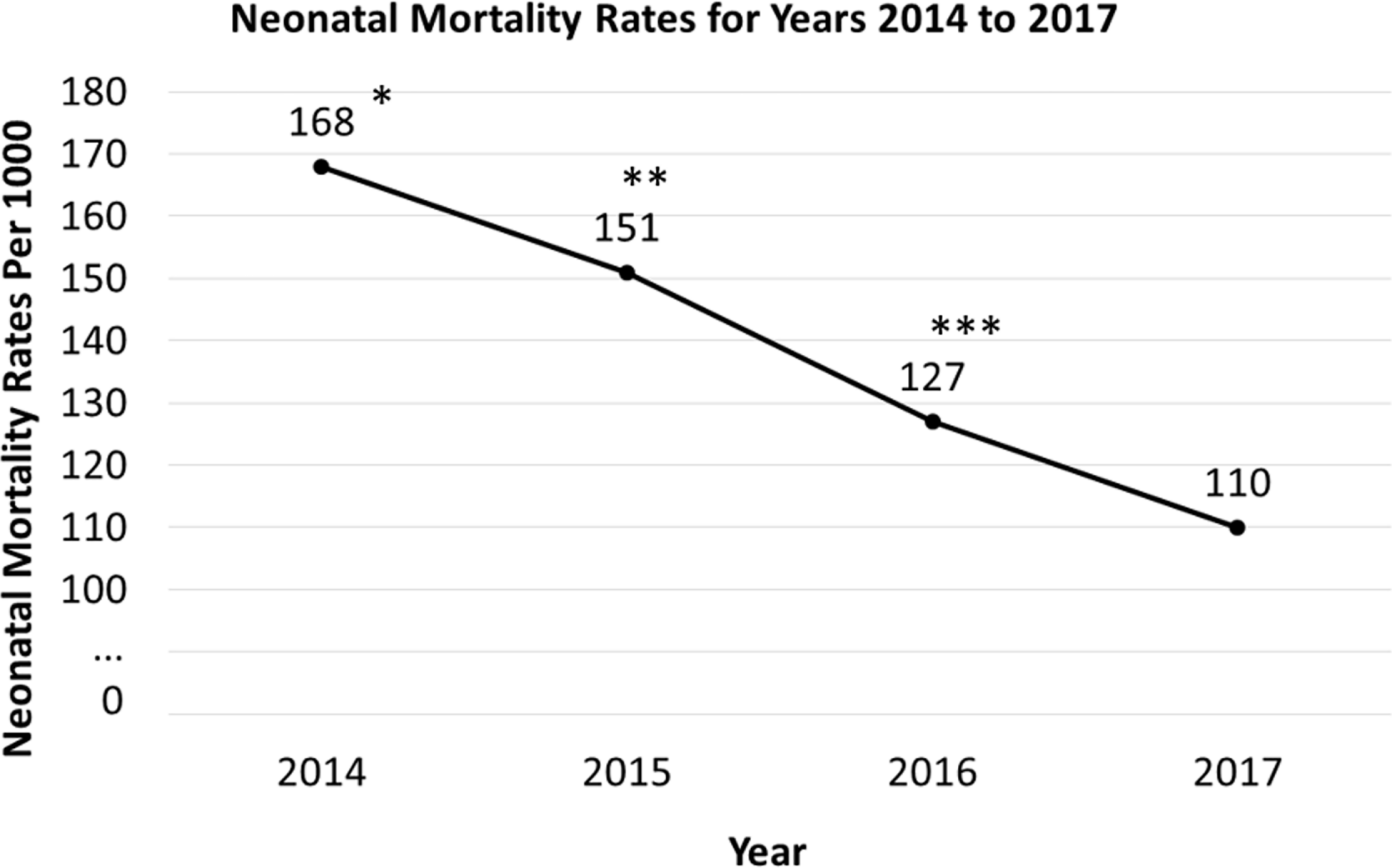
The progressive decrease in mortality from September 2014 through June 2017. *Comparing years 2014 and 2017—Relative Risk Reduction (RR) and 95% Confidence Interval (CI) 0.658 (0.45–0.96) p = 0.031, ** Comparing years 2015–2017—RR 0.84 (0.73–0.97) p = 0.016. *** Comparing 2016 and 2017 RR 0.91 (0.78–1.06) p = 0.22.

### Fresh stillbirths

FSB pre (n = 105) (162/1000) versus post implementation (n = 369) (111/1000) were of comparable BW, 1486±421 grams versus 1520± 438 grams (p = 0.51) but of lesser GA, 30.44 ± 2.0 weeks vs 30.97 ±2.1 weeks (p = 0.03). There were no differences with regard to source of admission, gender and mode of delivery.

## Discussion

The principal finding of this pilot implementation initiative incorporating a care-package strategy, was significant for an overall 26% reduction in NM ≤ 7 days, and an unanticipated 33% reduction in FSB rates in preterm infants 28 to 34 6/7 weeks EGA. By subgroup analysis there was no difference in NM for infants 28 to 30 6/7 weeks EGA, but a significant 45% reduction for infants of EGA 31 to 34 6/7 weeks following implementation. Neonatal characteristics associated with increased NM included lesser BW, lower GA and outborn deliveries, particularly in preterm infant 28 to 31 6/7 weeks. Interventions associated with reduced NM included the combined use of ACS and neonatal antibiotics (70%) ± maternal antibiotics (67%). ACS when used alone was associated with a non-significant increase in NM, and a combination of ACS and maternal antibiotics was associated with an increased risk of NM, an effect that was most pronounced in the larger preterm infant > 30 6/7 weeks GA. Infants who died had lower initial temperatures as compared to survivors. There was a progressive decrease in NM per year of implementation, which corresponded to an increase in medication use as well as a decrease in moderate hypothermia.

Implementation of the care bundle was uneven. Thus the use of ACS increased substantially at three hospitals. Although the increase in use was significant at Haydom, only 22% of mothers received ACS post implementation. This low percentage appears to be related to the fact that many mothers arrive at this hospital already in well-established labour. At KCMC there was no difference in ACS administration pre versus post implementation. We consider this the most plausible explanation for the apparent lack of effect of the care bundle on NM at this hospital. Given that many mothers may arrive in labour late, the ACS administration rate of approximately 68% (range 57 to 74%) at MNH, KCMC and BMC would seem to be an achievable goal at most hospitals. Regarding other components of the care bundle, maternal antibiotics increased substantially at all hospitals, although the rate was approximately 30% at BMC and Haydom. A potential explanation for the lower administration at BMC may reflect a maternal fee for antibiotic use at this hospital. A marked increase in use of neonatal antibiotics was noted at all hospitals. Finally, combination therapy increased more than two fold to 58% post implementation. This is relevant since combination therapy that included ACS and antibiotics was associated with a marked reduction in NM (range 67 to 70%). Temperature measurements increased significantly at all sites. Focus on maintaining temperature was accompanied by a three-fold reduction in moderate hypothermia post implementation.

These pilot observations are important and consistent with proof of concept, that interventions which are standard practice in developed countries, if implemented in a resource-limited setting, can significantly reduce NM. This is highly relevant for Tanzania and other resource limited countries, with minimal respiratory support other than oxygen supplementation, and the inability to routinely undertake a basic infectious workup. However, successful implementation is a complex issue influenced by many factor including BW, GA, temperature, delivery location, and adherence to the care bundle. The impact on NM was extremely favourable with combination therapy involving ACS and neonatal antibiotics ± maternal antibiotics. Conversely and surprisingly, a combination of ACS and maternal antibiotics was associated with increased mortality, an effect that was particularly prominent in the larger preterm infant 31 to 34 6/7 weeks GA. Potential factors contributing to this unanticipated observation remain unclear but important to elucidate. Both maternal and neonatal antibiotics when used singularly had no impact on NM. We speculate the benefit of combination therapy (ACS and antibiotics) on NM relates in part to the well-described positive impact on lung development and enhancement of pulmonary function with ACS. However, the pre-emptive approach to treating possible neonatal infection is likely an important contributing factor.

The administration of ACS to mothers of expectant preterm infants has long been the standard of care in the resource-replete setting and has consistently been associated with a reduction in NM in the preterm infant.[19] In the most recent Cochrane review, administration of ACS to mothers of preterm infants was associated with a significant reduction in NM and major morbidities, i.e. moderate/severe respiratory distress syndrome.[20] The authors concluded, “most evidence comes from high income countries and hospital settings; therefore, the results may not be applicable to low-resource settings with high rates of infections.” The data in this report support this hypothesis. Thus when used alone, ACS was associated with increased NM, consistent with the findings of Althabe, et al. [11] However, when ACS was combined with antibiotics there was a significant reduction in NM. Moreover there was a dose dependent positive effect of ANS on reducing NM. We consider these factors as the most likely explanation accounting for the differences in outcome between the Althabe and our study. [11, 14]

The necessity to avoid moderate hypothermia is a desired goal given that there is strong evidence of a dose dependent increase in mortality. [12,13] In this report, moderate hypothermia was almost two fold more likely to be present in those infants who died. Conversely, the finding that survivors were more likely to have temperatures in the normal range, may in part reflect the overall positive impact of ACS, including through an enhanced skin barrier. [21]

The care bundle preventative strategy was introduced with the goal of serving as a bridge linking labour and delivery (maternal antibiotics and ACS), resuscitation (HBB and thermal protection), the “Golden Hour” following delivery (continued thermal protection and early initiation of antibiotics), with the subsequent practice of providing essential newborn care (breast-feeding, eye-care, and continued management of infections). The “Golden Hour” is an increasing important concept in the resource-replete setting for improving extremely Preterm infant outcomes. [22]

The reasons contributing to the unanticipated reduction in FSB rates remain unclear. We speculate this may reflect an *in utero* effect of ACS on lung development. This in turn may have facilitated spontaneous respirations upon delivery, or in response to stimulation and suctioning in the non-breathing infant. However, FSB were of a lesser GA but comparable BW when comparing the pre- versus the post-implementation period. This observation will be important to trend as the rollout of the care bundle moves forth in Tanzania.

The study has several limitations. First, it was not randomized but rather a pilot implementation initiative. We chose this approach because we considered it unethical to perform a randomized study, given the overwhelming evidence, for a reduction in neonatal and major morbidities with ACS and antibiotics.[20] Second, the data collection form specifically developed for the care bundle, only became a mandatory part of record in September 2014. This limited the pre-implementation period for data collection. In this regard as noted previously, at KCMC approximately 50% of the mothers had received ACS during the pre-implementation period. Third, we were unable to determine the overall contribution of intrauterine growth restriction to the findings in this preterm population, in part because of a lack of relevant information in the data collection form. Fourth, since this was an evaluation of routine practice change and audit of outcomes, there is a chance that other (unrecorded) care practices also changed purely by coincidence. This could have introduced some potential for bias in the measurement of effect size.

In conclusion, a low cost care bundle and adherence to temperature control, relatively easy to administer by an obstetric provider, was associated with a significant reduction in preterm mortality, presumably by reducing respiratory morbidity and minimizing infection. If the findings can be replicated in other resource-limited settings, the ability to further reduce the less than 5 year mortality rates is enormous.

## Supporting information

S1 Data File (XLSX)

## Abbreviations

ACS: Antenatal Corticosteroid Treatment
BA: Birth Asphyxia
BMC: Bugando Medical Centre
BW: Birth Weight
CI: Confidence Interval
CPAP: Continuous Positive Airway Pressure
FSB: Fresh Still Birth
EGA: Estimated Gestational Age
HBB: Helping Babies Breathe
KCMC: Kilimanjaro Christian Medical Center
MA: Maternal Antibiotics
MNH: Muhimbili National Hospital
NA: Neonatal Antibiotics
NM: Neonatal Mortality
OR: Odds Ratio
RR: Relative Risk
WHO: World Health Organization
